# Detection of SENV Virus in Healthy, Hepatitis B- and Hepatitis C-Infected Individuals in Yazd Province, Iran

**DOI:** 10.7508/ibj.2016.03.006

**Published:** 2016-07

**Authors:** Sayedeh Azimeh Hosseini, Majid Bouzari

**Affiliations:** Department of Biology, Faculty of Science, University of Isfahan, Isfahan, Iran

**Keywords:** Hepatitis B virus, Hepatitis C virus, SEN virus

## Abstract

**Background:**

SEN virus (SENV) is the latest virus proposed as a cause of unknown hepatitis cases. Among nine detected genotypes of the virus, genotypes D and H are more frequent in hepatitis cases of unknown origin. The aim of this study was to determine the frequency of SENV-D and SENV-H genotypes in the sera of healthy individuals and hepatitis B and C patients.

**Methods:**

Totally, 200 serum samples from healthy individuals as well as 50 hepatitis B and 50 hepatitis C patients were collected. Anti-HCV (hepatitis C virus), anti-human immunodeficiency virus, hepatitis B surface antigen and anti-HBV (hepatitis B virus) core antigen were detected, and serum alanine aminotransferase (ALT) and aspartate aminotransferase (AST) were measured. Viral DNA was subjected to nested PCR. Fisher's exact and unpaired ANOVA tests were used for statistical analyses.

**Results:**

SENV was detected in 90%, 66%, and 46% of the healthy individuals HBV and HCV-positive individuals, respectively. The frequency of SENV and its two genotypes were significantly lower in hepatitis B and hepatitis C patients (*P*<0.01). Also, the frequency of SENV-H was higher than SENV-D in all studied groups. In SENV-positive HBV patients, the level of ALT and AST enzymes were significantly less than SENV-negative patients (*P*<0.05). It was the same for SENV-H-negative and -positive cases.

**Conclusions:**

The levels of liver enzymes were significantly lower in HBV patients co-infected with SENV compared to HBV patients (*P*<0.05), indicating a positive impact of the virus in liver pathology by decreasing liver damage and thus decreasing the liver enzymes.

## INTRODUCTION

Over 80% of hepatitis cases are caused by five types of hepatitis viruses (A-E), which usually known as hepatotropic viruses^[^^1^^]^. The etiology of the remaining 20% cases and also 10% transfusion-associated hepatitis are unknown, which may be due to the involvement of other viruses^[^^2^^]^. The latest virus proposed as a cause of this kind of hepatitis (non-A-E) is SEN virus (SENV). SENV was first identified in the serum of a *human immunodeficiency virus* (HIV) co-infected patient with hepatitis of unknown etiology in Italy in 1999^[^^3^^-^^5^^]^. It is a circular ssDNA non-enveloped virus of about 26 nm consisting at least three open reading frames^[^^2^^,^^3^^]^. This virus has been classified as an unassigned virus of Anelloviridae^[^^6^^]^. For SENV, nine genotypes (A-I) have been detected; among which genotypes D and H are more frequent in individuals infected with hepatitis of unknown origin compared to other genotypes^[^^3^^,^^7^^]^.

As no correlation has been reported between liver pathology and SENV infection^[^^2^^]^, this study attempted to determine the frequency of SENV-D and SENV-H genotypes of SENV in the sera of healthy individuals and hepatitis B and hepatitis C patients. Liver enzymes, alanine amino-transferase (ALT) and aspartate aminotransferase (AST), were determined in hepatitis patients to reveal the possible impact of the virus on the disease.

## MATERIALS AND METHODS

In total, 200 blood sera from healthy individuals as well as 50 hepatitis B and 50 hepatitis C patients were collected in Yazd from June to November 2012. The samples were stored at -20°C until use. The cases included 148 males and 152 females. The protocol for the research project was approved by Ethics Committee of the University of Isfahan (Isfahan, Iran) and conformed to the provisions of the Declaration of Helsinki (as revised in Tokyo 2004).

**Laboratory studies**

Antibodies to hepatitis C virus (anti-HCV) and Anti-HIV, hepatitis B surface antigen and antibodies to hepatitis B virus (HBV) core antigen were measured by a commercial ELISA kit (Acon laboratories Inc., USA) according to manufacturer's guidelines. ALT and AST measurements were determined using a laboratory test kit (Pars Lab., Iran).

**Viral DNA extraction**

Viral DNA was extracted from 220 μl serum samples. Sera were mixed with 6.5 μl 0.25% SDS and 10 μl 0.2 M NaCl and then proteinase K solution (10 mg/ml) was added. Next, the mixture was incubated at 65°C for 2 h. Two phenol-chloroform extraction steps, followed by a chloroform treatment were applied to eliminate proteins. DNA was precipitated using cold ethanol (100%) (Merck, Germany) and was dissolved in distilled dionized water (50 μl)^[^^3^^]^.

**Detection of SENV DNA**

SENV DNA was detected by polymerase chain reaction (PCR) with SENV-specific primers according to Kojima *et al*. ^[^^7^^]^. In the first round of PCR, a primer pair, which detected a 349-bp conserved region for all SENV genotypes (A-I), was used (forward primer AI-1F **[**5-TWCYCMAACGACCAGCTAGACCT-3] and reverse primer AI-1R [5-GTTTGTGGTGAGCAGAA CGGA-3]). In the second round of PCR, the following forward and reverse primers were used: D-1148F (5-CTAAGCAGCCCTAACACTCATCCAG-3) and D-1341R (5-GCAGTTGACCGCAAAGTTACAAGAG-3) for SENV-D with a sequence length of 198 bp and H-1020F (5-TTTGGCTGCACCTTCTGGTT-3) and H-1138R (5-AGAAATGATGGGTGAGTGTTAGGG-3) for SENV-H with a sequence length of 124 bp. In the first round, PCR mixture volume of 20 μl was consisted of 0.5 pmol/μl each primer (AI-1F and AI-1R), 50 mM KCl, 20 mM Tris-HCl (pH 8.4), 2.5 mM MgCl_2_, 200 μM each dNTP, 1 U Smart Taq DNA polymerase (Cinnagen, Iran), and 2 μl extracted DNA. PCR amplification was performed with a denaturation step of 94°C for 2 minutes and a set of 30 cycles (94°C for 30 seconds, 55°C for 30 seconds and 72°C for 2 minutes) with a final extension of 10 minutes at 72°C in a thermo cycler gradient (Eppendorf/5331, Germany). One microliter of first-round PCR products was used in the second round. The second-round PCR involved a denaturation step at 94°C for 2 min and 30 cycles (94°C for 30 seconds, 56°C for 30 seconds, and 72°C for 2 minutes) for both SENV-D and SENV-H with a 10-min final extension at 72ºC. All of the amplified products were electrophoresed and analyzed on 1% agarose gel.

**DNA sequencing**

Two PCR products of each group of healthy individuals as well as hepatitis B and hepatitis C patients were selected randomly and sequenced (Takapou Zist Ltd., Iran). The sequences were aligned using WU-BLAST2 search of the determined sequences against a nucleotide sequence database (EMBL, European Bioinformatics Institute). Multiple sequence alignments were performed using ClustalW in MEGA5 software (Molecular Evolutionary Genetics Analysis software, version 5.10)^[^^8^^]^ and Bio Edit software (Bio Edit sequence alignment sequence, version 7.1.11)^[^^9^^]^. Neighbor-joining method was used to construct a phylogenetic tree based on the partial ORF1 of the obtained sequences with accession numbers of AB856066 (SENV-H-positive HBV patients) (AH1), AB856067 (SENV-D-positive HBV patients) (AH2), AB856068 (SENV-H-positive HCV patients) (AH3), AB856069 (SENV-D-positive HCV patients) (AH4), AB856071 (SENV-D-positive healthy individuals) (AH5), and AB856070 (SENV-H-positive healthy individuals) (AH6) against some known sequences obtained from the GeneBank.

**Statistical analyses**

Fisher's exact and unpaired ANOVA tests were used for statistical analyses using GraphPad Software Prism 5.04.

## RESULTS

The frequency of SENV infection in healthy individuals and hepatitis B and hepatitis C patients is shown in Table 1. SENV was detected in 90% of the healthy individuals (181 out of 200) as well as in 66% of the HBV- (33 out of 50) and in 46% of the HCV-positive individuals (23 out of 50). The frequency of SENV and its two genotypes were significantly low in both hepatitis B and hepatitis C patients (*P*<0.05).

A comparison of SENV genotype frequencies in SENV-positive cases is shown in Table 2. In all studied groups, the frequency of SENV-H was higher than SENV-D. Also, the comparison of gender, age and liver enzymes in SENV-positive and -negative healthy individuals indicated that there are no significant differences between these two groups (*P*>0.05) (Table 3). As shown in Table 4, the levels of AST (SGOT) and ALT (SGPT) enzymes in SENV-positive HBV patients were significantly lower than SENV-negative patients (*P*<0.05). Also, this result was the same for SENV-H-negative and -positive cases. The frequency of SENV (total) and SENV-H infection was significantly higher in male than female HCV patients (*P*<0.05) (Table 4). 

**Table 1:** The frequency of SENV and its two genotypes in hepatitis B and hepatitis C patients and healthy individuals

**Study group**	**Total**	**SENV ** **positive**		**SENV-D positive**		**SENV-H positive**		**Both ** **SENV-D & SENV-H**	
		**F**	**%**	**F**	**%**	**F**	**%**	**F**	**%**
Healthy individuals	200	181	90.5	82	41	174	87	74	38
Patients total	100	56	56.0	25	25	51	51	18	18
HCV patients	50	23	46.0	12	24	19	38	6	12
HBV patients	50	33	66.0	13	26	32	64	12	24
*P* value healthy vs. HBV	-	<0.0001*		0.052		<0.0004*		0.1323	
*P* value healthy vs. HCV	-	<0.0001*		0.0333*		<0.0001*		0.0006*	
*P* value HBV vs. HCV	-	0.06		1.00		0.0159*		0.192	
*P* value healthy and patients		<0.0001*		0.0072*		<0.0001*		0.0008*	

* Significant difference; F, frequency, HCV, hepatitis C virus; HBV, hepatitis B virus

**Fig. 1:**
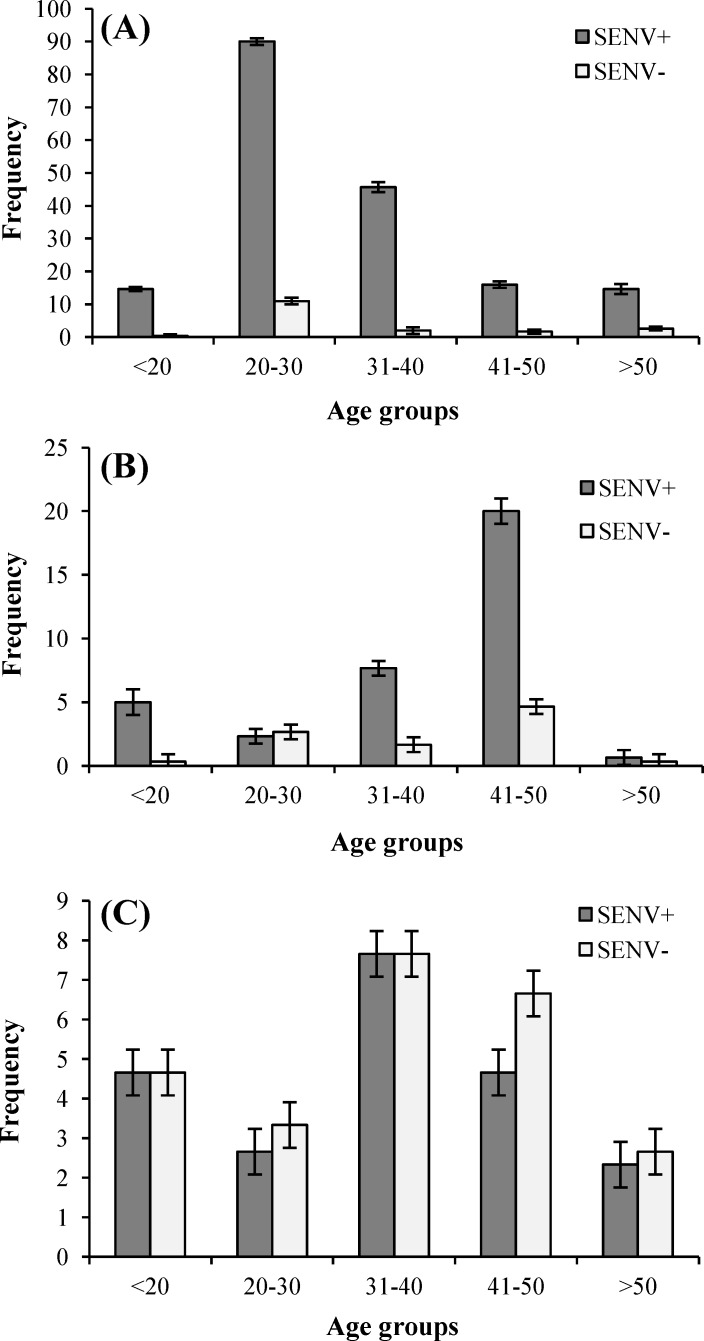
The comparison of frequency of infection with SENV in different age groups of (A) healthy, (B) HBV- and (C) HCV-infected individuals. In healthy individuals, the frequency of infection in age group of 20-30 years was significantly higher than other groups (*P*<0.05). In HCV- and HBV-infected individuals, no significant differences were observed in different age groups. Bars=standard error (SE

As shown in Figure 1, in healthy individuals, the frequency of infection in the age group of 20-30 years old was significantly higher than that in other groups (*P*<0.05). However, in HCV- and HBV-infected individuals, the frequency of infection in the age groups of 31-40 and 20-30 years old was higher compared to other age groups, and the differences were not significant (*P*>0.05). In addition, in age group of 20-30 years, the frequency of infection in HBV patients was significantly higher than HCV patients (*P*<0.05), and in healthy individuals, it was significantly higher than HCV patients in all age groups (*P*<0.05). This result was the same for HBV patients but only in the age group of 41-50, and the difference was significant (*P*<0.05).

The first round of PCRs using the following primer pairs showed the expected amplification products of 349 bp (AI-1F: 5-TWCYCMAACGACCAGCTAGAC CT-3 and AI-1R: 5-GTTTGTGGTGAGCAGAACGG A-3).

In addition, the second round of PCRs using the following primers could detect both 198 and 124 bp products for SENV-D and SENV-H, respectively. D-1148F: 5-CTAAGCAGCCCTAACACTCATCCAG -3 and D-1341R: 5GCAGTTGACCGCAAAGTTACA AGAG-3) for SENV-D and H-1020F: 5-TTTGGCTGC ACCTTCTGGTT-3 and H-1138R: 5-AGAAATGATG GGTGAGTGTTAGGG-3 for SENV-H. 

**Fig. 2:**
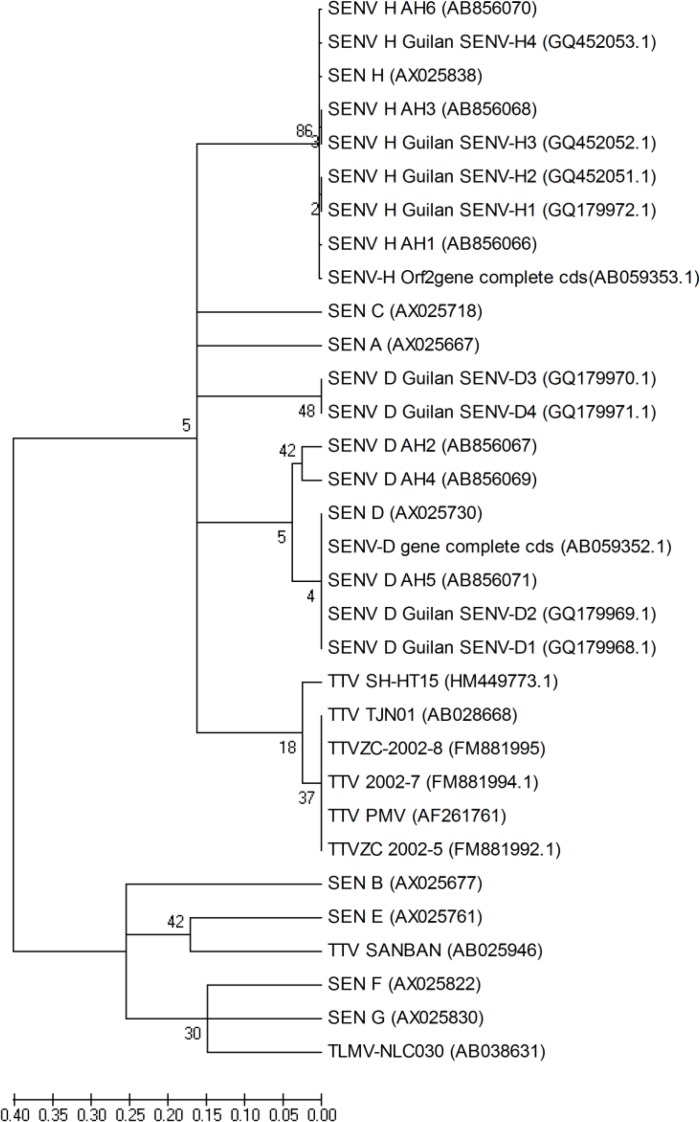
Phylogenetic tree constructed by neighbor-joining method based on our sequences with accession numbers of AB856066 (SENV-H-positive HBV patients) (AH1), AB856067 (SENV-D-positive HBV patients) (AH2), AB856068 (SENV-H-positive HCV patients) (AH3), AB856069 (SENV-D-positive HCV patients) (AH4), AB856071 (SENV-D-positive healthy individuals) (AH5), and AB856070 (SENV-H-positive healthy individuals) (AH6) against some sequences from the GeneBank. SENV-D sequences detected in this study and some of the sequences of the viruses from Guilan, Iran are placed in two distinct clusters

As shown in Figure 2, a high genomic homology was found between the sequences of SENV-H obtained in this study and those of SENV isolated from Northern Iran, Guilan and SENV-H isolates from elsewhere. Although SENV-D sequences had a high homology with the sequences of the viruses from Guilan, some of them were placed in a distinct cluster.

## DISCUSSION

Totally, SENV was detected in 56% of the hepatitis B and hepatitis C patients, which were significantly lower than that of healthy individuals (90.5%) tested. 

**Table 2:** The frequency of SENV genotypes in SENV-positive cases

**Genotypes**	**HBV & HCV patients ** **(n=56)**		**Healthy individuals ** **(n=181)**		***P*** **value**
	**F**	**%**	**F**	**%**	
SENV-D	25	40.64	82	54.30	1.000
SENV-H	51	91.07	174	96.13	0.160
SENV-H and D	18	32.14	74	40.80	0.270

F, frequency, HBV, hepatitis B virus; HCV, hepatitis C virus

**Table 3:** The comparison of gender, age and liver enzymes in SENV-positive  and -negative healthy individuals

**Variable**	**SENV** **positive**	**SENV** **negative**	***P*** **value**
Male/female	77/105	4/14	0.1315
Age	35.39±4.062	31.66±0.9012	0.2358
AST (SGOT)	22.79±1.096	18.40±0.7630	0.1662
ALT (SGPT)	30.08±3.063	23.20±4.117	0.4478

AST, aspartate amino transferase; ALT, alanine transaminase; SGOT, serum glutamic oxaloacetic transaminas; SGPT, serum glutamic-pyruvic transaminase

This result is contrary to Ghasemi Dehkordi and Doosti's report^[^^10^^]^ from Chaharmahal and Bakhtiari Province of Iran, who showed the frequency of virus was higher in HBV- and HCV-infected individuals. It is also in contrast to the reports from other countries^[^^11^^-^^17^^]^.

The frequency of SENV infection detected in this study is similar to that reported by Karimi-Rastehkenari and Bouzari^[^^3^^]^ from Guilan Province of Iran. This result confirms the higher prevalence of the virus in healthy individuals in Iran compared to other countries^[^^11^^,^^13^^,^^15^^-^^19^^]^. The lower frequency of infection reported in Tehran, Iran (23%)^[^^20^^]^ could be correlated to the methodology used. The frequency of SENV-H in HBV and HCV patients and healthy individuals was higher compared to SENV-D. In the case of healthy individuals, the results were the same as what already reported from Iran^[^^3^^,^^20^^]^ as well as from Turkey^[^^2^^,^^15^^]^ and Taiwan^[^^11^^]^. However, it was in contrast with the reports from Egypt^[^^4^^]^, which detected SENV-D in all SENV positives of control group (100%)^[^^4^^]^ and from Japan^[^^21^^]^, which demonstrated SENV-D in 77% and SENV-H in 15% of the individuals^[^^21^^]^.

In the present study, no significant correlation was observed (*P*>0.05) in the mean age of the HBV/HCV patients and healthy individuals for SENV-positive and -negative cases, which was in agreement with the reports from Mikuni *et al*.^[^^12^^]^, Serin *et al*.^[^^15^^]^, Pirovano *et al*.^[^^19^^]^, and Yoshida *et al*.^[^^22^^]^. Also, the frequency of SENV infection in men and women in healthy individuals and HBV patients were identical. However, the frequency of SENV infection of men with HCV infection was significantly higher than women (*P*<0.05), which is in agreement with the finding of Schreter *et al*.^[^^14^^]^ and Chiou *et al*.^[^^23^^]^.

A very high statistical association of SENV-D and SENV-H with non-A-E hepatitis cases has already been reported^[^^17^^]^, and the possible role of SENV as an additional factor has been proposed. Notably, in current study, the levels of liver enzymes (ALT and AST) were significantly lower in HBV patients co-infected with SENV (*P*<0.05) compared to HBV patients. This issue may indicate the positive impact of the virus on liver pathology by decreasing liver damage, which in turn can result in reduction of liver enzyme levels.

**Table 4:** The comparison of gender, age, and liver enzymes in SENV (genotypes)-positive and -negative HBV and HCV patients

**Variable**	**Virus**	**SENV**		***P*** **value**	**SENV-H**		***P*** **value**	**SENV-D**		***P*** **value**
		**Positive**	**Negative**		**Positive**	**Negative**		**Positive**	**Negative**	
Male/female	HBV	15/18	12/5	0.135	14/18	13/5	0.077	8/5	19/18	0.74
	HCV	23/0	19/10	0.008*	19/0	21/10	0.008*	10/2	30/8	1.000
Age	HBV	32.56±2.00	42.00±4.21	0.044	32.47±2.52	41.67±3.99	0.04	36.8±3.15	35.33±2.06	0.64
	HCV	36.83±2.49	36.44±2.50	0.915	36.26±2.848	36.84±2.26	0.87	37.8±3.15	36.33±2.06	0.7
AST (SGOT)	HBV	22.20±2.093	34.73±4.46	0.006*	22.20±2.093	34.73±4.46	0.006*	26.50±4.81	27.03±2.67	0.921
	HCV	54.90 ±10.0	68.41±30.27	0.6481	49.06±8.021	71.65±26.58	0.44	71.00±19.62	57.83±18.03	0.703
ALT (SGPT)	HBV	27.23±5.168	56.33±12.60	0.017*	27.23±5.168	56.33±12.6	0.017*	35.55±11.7	38.73±7.10	0.817
	HCV	51.73±9.73	61.60±32.06	0.7607	52.50±11.71	59.38±26.68	0.83	62.10±15.26	54.66±20.43	0.844

* Significant difference; AST, aspartate amino transferase; ALT, alanine transaminase; SGOT, serum glutamic oxaloacetic transaminas; SGPT, serum glutamic-pyruvic transaminase; hepatitis B virus; HCV, hepatitis C virus

Although SENV-D sequences had a high homology with sequences of the viruses from Guilan, Iran, some of them were placed in a distinct cluster, which may demonstrate the presence of at least two different subgroups in Iran.

Collectively, compared to other reports, the frequency of SENV in hepatitis B and hepatitis C patients were significantly lower than healthy individuals tested. The frequency of SENV-H was higher than SENV-D in HBV and HCV patients and healthy individuals. No significant correlation was observed in the mean age of the HBV/HCV patients and healthy individuals in SENV-positive and -negative cases. The frequency of SENV infection in men and women in healthy individuals and HBV patients were identical. In addition, the levels of liver enzymes were significantly lower in HBV patients co-infected with SEN virus as compared to HBV patients. This may indicate a positive impact of the virus on liver pathology by decreasing the liver damage, followed by a decline in liver enzymes levels. The Phyolgenetic tree indicated the possibility of the presence of at least two different subgroups in Iran.
